# Blood‐based biomarkers for Alzheimer's disease

**DOI:** 10.15252/emmm.202114408

**Published:** 2021-12-03

**Authors:** Antoine Leuzy, Niklas Mattsson‐Carlgren, Sebastian Palmqvist, Shorena Janelidze, Jeffrey L Dage, Oskar Hansson

**Affiliations:** ^1^ Clinical Memory Research Unit Department of Clinical Sciences Lund University Malmö Sweden; ^2^ Department of Neurology Skåne University Hospital Lund Sweden; ^3^ Wallenberg Centre for Molecular Medicine Lund University Lund Sweden; ^4^ Memory Clinic Skåne University Hospital Lund Sweden; ^5^ Stark Neuroscience Research Institute Indiana University School of Medicine Indianapolis IN USA

**Keywords:** Alzheimer, Aβ, biomarkers, blood, P‐tau, Biomarkers, Neuroscience

## Abstract

Neurodegenerative disorders such as Alzheimer's disease (AD) represent a mounting public health challenge. As these diseases are difficult to diagnose clinically, biomarkers of underlying pathophysiology are playing an ever‐increasing role in research, clinical trials, and in the clinical work‐up of patients. Though cerebrospinal fluid (CSF) and positron emission tomography (PET)‐based measures are available, their use is not widespread due to limitations, including high costs and perceived invasiveness. As a result of rapid advances in the development of ultra‐sensitive assays, the levels of pathological brain‐ and AD‐related proteins can now be measured in blood, with recent work showing promising results. Plasma P‐tau appears to be the best candidate marker during symptomatic AD (i.e., prodromal AD and AD dementia) and preclinical AD when combined with Aβ42/Aβ40. Though not AD‐specific, blood NfL appears promising for the detection of neurodegeneration and could potentially be used to detect the effects of disease‐modifying therapies. This review provides an overview of the progress achieved thus far using AD blood‐based biomarkers, highlighting key areas of application and unmet challenges.

## Graphical abstract

This Review discusses recent advances in blood‐based biomarkers for Alzheimer's disease, highlighting the key areas of application and unmet challenges.

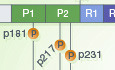

**Glossary**
Amyloid PET: Aβ‐specific ligands for use with positron emission tomography
Antibodies: immune proteins produced in response to an antigen
Area under the receiver operating curve: a measure of the ability of a classifier to distinguish between positive and negative classes (i.e., normal and abnormal)
Aβ plaques: abnormal extracellular deposits of the Aβ peptide
Biomarkers: a biomarker is an objectively measurable parameter that can be treated as an indicator of biological processes or responses to a treatment
Enzyme‐linked immunosorbent assays: though various ELISA variants all are characterized by the following elements: an antigen, one or several antibodies specific to that antigen, and a system to quantify the amount of antigen present
Head‐to‐head study: a study design in which two methods are directly compared using data from the same individuals
Immunomagnetic reduction platforms: a technique in which the concentration is measured by comparing changes in magnetic responses between free and conjugated magnetic nanoparticles
Immunoprecipitation mass spectrometry assay: an assay combining immunoprecipitation and mass spectrometry. Using this approach, desired analytes are first selectively captured from solution prior to analysis with mass spectrometry
Mild cognitive impairment: a state of cognitive impairment intermediate between those due to normal aging and dementia (i.e., objective cognitive deficits beyond that expected given their age and education yet of insufficient severity to meet criteria for dementia)
Neurofibrillary tangles: abnormal intracellular accumulations of the tau protein
Neuroinflammation: astrocytic and microglial activation
Preanalytical variables: these include tube type and time from blood collection to centrifugation and pipetting of the plasma
Sensitivity: the ability of a test to correctly identify patients with a disease
Single‐molecule array: a digital assay technique allowing for the measurement of single‐molecule immunocomplexes
Specificity: the ability of a test to correctly identify people without the disease
Tau PET: tau‐specific ligands for use with positron emission tomography

## Introduction

Neurodegenerative disorders such as Alzheimer's disease (AD) are the leading causes of dementia and carry immense social and economic costs. Worldwide, an estimated 50 million people currently live with dementia, with this figure projected to exceed 80 million by 2030 (Prince *et al*, 2016). With increasing age as the greatest risk factor for dementia, a driving factor behind these rising prevalence figures is increased longevity (Winblad *et al*, 2016); as such, these disorders represent a major and increasing global health challenge.

On clinical grounds alone, the differential diagnosis of AD can prove challenging, even for dementia experts (Beach *et al*, 2012; Salloway *et al*, 2014). Accurate prognosis and disease monitoring are also difficult when relying on clinical information only. As a result, biomarkers have come to play an increasingly important role in the field. Providing an objective measure of relevant pathophysiology *in vivo*, biomarkers are now included in modern research diagnostic criteria for AD (Dubois *et al*, 2007, 2014; Albert *et al*, 2011; Jack *et al*, 2011, 2018; McKhann *et al*, 2011; Sperling *et al*, 2011a) and are recommended for use in clinical trials by regulatory agencies (Hampel *et al*, 2010). Further, the use of biomarkers is important in the context of treatment, both in terms of ensuring that AD patients access available symptomatic treatments and in terms of providing an accurate prognosis early on in the disease course should disease‐modifying therapies become available for AD (Abbasi, 2018). The AD biomarker research field is now moving from studies of group‐level associations to subject‐level diagnosis and prognosis in real‐world scenarios. Examples of this include recent work aiming to assess the risk for cognitive decline using biomarkers in patients with mild cognitive impairment (MCI) (van Maurik *et al*, 2017, 2019a, 2019b; Cullen *et al*, 2021a). However, a substantial irreversible neuronal loss can already be seen by this stage—which may reduce the likelihood of disease‐modifying therapies to prevent dementia onset (Sperling *et al*, 2011b). There is therefore now an increasing focus also on cognitively unimpaired (CU) older individuals at risk for progression to AD dementia on the basis of biomarker evidence of brain AD pathology (Sperling *et al*, 2014; Cullen *et al*, 2021b).

Due to it being in direct contact with the central nervous system, cerebrospinal fluid (CSF) has proven an ideal source of information for the detection and measurement of biochemical abnormalities within the brain (Hampel *et al*, 2012); examples include CSF amyloid‐β (Aβ) 42—alone, and in ratio with Aβ40 (Aβ42/Aβ40)—reflecting Aβ deposition; phosphorylated tau (P‐tau), reflecting tau pathology; and neurofilament light (NfL) (Khalil *et al*, 2018), reflecting neurodegeneration. These abnormalities can also be measured using positron emission tomography (PET) with compounds (tracers) specific for Aβ, tau, and synaptic impairment. The global use of CSF and imaging‐based biomarkers remains limited owing to the perceived invasiveness of lumbar punctures and the high cost and low availability of PET imaging (Duits *et al*, 2016). This has led to a growing interest in the use of blood‐based biomarkers, with recent work showing promising results (Nakamura *et al*, 2018; Palmqvist *et al*, 2019b; Schindler *et al*, 2019; Janelidze *et al*, 2020a; Karikari *et al*, 2020b, 2021). In the present review, we aim to provide an overview of the progress achieved thus far using AD blood‐based biomarkers and to highlight key areas of application and remaining challenges.

## Markers of Aβ pathology (Aβ42/Aβ40)

Following the discovery of multiple C‐terminal forms of Aβ, the 42‐amino acid isoform of Aβ (Aβ42) was found to be highly aggregation‐prone and predominant in diffuse and cored plaques in AD (Iwatsubo *et al*, 1994; Tamaoka *et al*, 1994). Using enzyme‐linked immunosorbent assays (ELISAs) specific to Aβ42, a marked reduction in CSF Aβ42 was seen in AD (Motter *et al*, 1995; Blennow & Hampel, 2003), with levels shown to correlate inversely with cortical plaque load at post‐mortem (Strozyk *et al*, 2003; Tapiola *et al*, 2009) and in biopsy studies (Seppala *et al*, 2012). Combining Aβ42 with Aβ40 (using a ratio) corrects inter‐individual differences in Aβ processing and possible preanalytical confounders and increases concordance with amyloid PET (Hansson *et al*, 2019).

### Assays

Plasma Aβ assays include ELISA‐based immunoassays on the Luminex xMAP (Hansson *et al*, 2010), single‐molecule array (Simoa) (Janelidze *et al*, 2016), Elecsys (Palmqvist *et al*, 2019b), and immunomagnetic reduction (IMR) platforms as well as immunoprecipitation mass spectrometry (IP/MS) assays (Pannee *et al*, 2014; Ovod *et al*, 2017; Schindler *et al*, 2019). For the IP/MS assays, plasma Aβ is enriched via immunoprecipitation with Aβ antibodies coated onto paramagnetic beads. These antibodies are either directed to the mid‐region (Ovod *et al*, 2017; Nakamura *et al*, 2018) or N‐terminal (Pannee *et al*, 2014) part of Aβ. Stable isotope‐labeled synthetic Aβ peptides (e.g., Aβ42 and Aβ40) are then used as mass spectrometry quantification standards. In the study by Nakamura *et al*, however (Nakamura *et al*, 2018), Aβ38 was used as a single stable isotope‐labeled standard for all Aβ isoforms.

Early work using Luminex xMAP technology in plasma failed to replicate the observed decrease of Aβ42 seen in CSF (Song *et al*, 2011; Toledo *et al*, 2013; Rembach *et al*, 2014; Swaminathan *et al*, 2014; Olsson *et al*, 2016), likely due to the use of clinical diagnosis as the standard of truth, analytical limitations inherent to these methods (e.g., epitope masking by hydrophobic Aβ peptides (Kuo *et al*, 1999)) and, possibly, to peripheral tissues contributing to the global pool of plasma Aβ (Li *et al*, 1998; Kuo *et al*, 2000; Roher *et al*, 2009; Hansson *et al*, 2010). In 2016, however, using a Simoa assay for Aβ—a technique allowing for the reduction of matrix effects via predilution of samples due to its very high analytical sensitivity (Rissin *et al*, 2010, 2011; Zetterberg *et al*, 2011)—plasma Aβ was found to be reduced in AD compared to controls and patients with MCI and vascular dementia and to separate abnormal from normal amyloid PET scans with moderate accuracy (AUC between 0.62 and 0.68) (Janelidze *et al*, 2016; Verberk *et al*, 2018). Higher AUCs have since been reported using a modified version of the Simoa assay with different antibodies (Verberk *et al*, 2020b). Simoa studies were followed by several studies using IP/MS (Ovod *et al*, 2017; Nakamura *et al*, 2018; Schindler *et al*, 2019). Using amyloid PET status as outcome, plasma Aβ42/Aβ40 showed high accuracy in CU individuals (Schindler *et al*, 2019) and across CU individuals and patients with mild‐to‐moderate AD (AUCs of between 0.84 and 0.97) (Ovod *et al*, 2017; Nakamura *et al*, 2018).

In a recent study that compared several IP/MS assays and immunoassays using a head‐to‐head design (Janelidze *et al*, 2021), certain IP/MS methods were shown to have superior performance to other IP/MS methods and all immunoassays using CSF Aβ42/40 and Aβ‐PET status as outcome. Though promising and highlighting the potential of plasma Aβ as an AD biomarker, IP/MS‐ and Simoa‐based studies are comparatively costly and require extensive development before they can be used in primary care or in screening large numbers of participants for AD clinical trials. Although this recent progress with high precision plasma Aβ measures has resulted in commercially available lab‐developed blood tests for the detection of AD pathology, fully automated, high‐throughput, and highly reliable analysis methods would facilitate implementation more broadly in clinical practice. Indeed, in the study by Janelidze *et al* (2021) comparing IP/MS and immunoassays, the Elecsys immunoassays (Roche Diagnostics) (Hansson *et al*, 2018) showed the numerically highest AUC (0.740). This is likely due to the immunoassays being fully automated and having very high analytical reliability and precision. Additional work using the Elecsys immunoassays for plasma Aβ42/Aβ40 showed that subjects could be differentiated based on their Aβ status with an AUC of 0.80 (Palmqvist *et al*, 2019b). The addition of *APOE* ε4 status—and, to a lesser extent, T‐tau and NfL—increased the AUC significantly to around 0.85–0.87, though accuracy was lower compared to those reported in the IP/MS studies (Ovod *et al*, 2017; Nakamura *et al*, 2018; Schindler *et al*, 2019).

In a recent study, however, a head‐to‐head comparison of plasma Aβ42/Aβ40 quantified with commercially available ELISA kits (EUROIMMUN) and prototype SIMOA assays (Amyblood; ADx NeuroSciences) (De Meyer *et al*, 2020) that used the same sets of monoclonal detector and capture antibodies showed that both provided identical accuracy for detecting amyloid PET status in a cohort of nondemented elderly individuals. The superior performance of these novel ELISAs can be attributed to technological advancements, including the use of C‐ and N‐terminal antibodies (Pesini *et al*, 2012), improved conjugation method (Cirrito *et al*, 2003; Lopez *et al*, 2008), and an improved understanding of the effects of preanalystical variables (Lachno *et al*, 2009). While head‐to‐head comparisons are required between the different ELISAs, their improved performance carries potentially important implications due to their being much more widely available than Simoa. Recently, novel ready‐to‐use Simoa‐based immunoassays (“Amyblood”) were developed to detect full‐length Aβ_1–42_ and Aβ_1–40_ (Thijssen *et al*, 2021b), with the Amyblood Aβ42/Aβ40 ratio showing technical and clinical performance comparable to the Quanterix triplex and Euroimmun ELISAs but superior specificity and selectivity than the Quanterix triplex kit.

A major limitation of plasma Aβ42/40, however, is that its levels are only decreased by 10–20% in individuals with cerebral Aβ pathology, compared to 40–60% for CSF Aβ42/40 (Nakamura *et al*, 2018; Verberk *et al*, 2018; Palmqvist *et al*, 2019a, 2019b; Schindler *et al*, 2019). This is likely due to plasma Aβ levels being affected by Aβ metabolism outside the brain (Li *et al*, 1998; Kuo *et al*, 2000; Roher *et al*, 2009; Hansson *et al*, 2010). As a result, plasma Aβ42/40 levels can be affected by small measurement variations caused by preanalytical handling (such as tube type and time from blood collection to centrifugation and pipetting of the plasma) and analytical performance (Rozga *et al*, 2019). This, in turn, can affect subject‐level classification (i.e., negative or positive for Aβ pathology). Given the more robust changes seen for Aβ42/40 in CSF—as well as the robustness of this measure to the interfering effects of preanalytical factors (Hansson *et al*, 2019)—CSF Aβ42/40 has overall shown a higher diagnostic accuracy than plasma Aβ42/40 and is less susceptible to variations in its optimal cut‐point (Schindler *et al*, 2019). Possibly, combining plasma Aβ42/40 with P‐tau or GFAP using an algorithm may make plasma Aβ42/40 more robust to preanalytical factors; however, this has not yet been studied.

### Differential diagnosis of AD dementia

Thus far, only one study has examined the ability of plasma Aβ42/Aβ40 to differentiate AD dementia from non‐AD dementia disorders (Palmqvist *et al*, 2020). Using immunoassays, however (Euroimmun ELISAs)—as opposed to mass spectrometry methods—the study reported poor diagnostic accuracy (AUC of 0.62) when using clinically diagnosed participants (AD dementia [*n* = 121] vs a non‐AD group [*n* = 99] including 45 patients with Parkinson's disease (without or with dementia) or multiple system atrophy, 21 with progressive supranuclear palsy or corticobasal syndrome, 12 with vascular dementia, and 21 with behavioral variant frontotemporal dementia or primary progressive aphasia). The AUC of plasma Aβ42/Aβ40 was higher, however, when using neuropathologically confirmed cases (AUC of 0.72 for intermediate to high likelihood of AD [*n* = 34] vs non‐AD [*n* = 47], where primary neuropathological diagnoses included PART (seven possible, 11 definite), 13 PD, three PSP, two VaD, three with white matter changes due to infarcts, one ALS, one multiple sclerosis, one showing diffuse astrocytoma, two CBD, one FTLD with TDP‐43 pathology, and two with NFT predominant dementia) (Palmqvist *et al*, 2020).

### Prediction of AD dementia and cognitive decline in MCI

Varying discriminative performance has been seen when differentiating MCI patients who converted to AD dementia from those that did not using plasma Aβ42/Aβ40 adjusted for age (AUC of 0.67) (Simren *et al*, 2021) and age, sex, education, and baseline MMSE (AUC ranging from 0.66 to 0.86 depending on cohort and assay) (Cullen *et al*, 2021a) (Table EV1). In a prospective study examining plasma Aβ42/Aβ40 and the risk of conversion from amnestic MCI to AD dementia, however, plasma Aβ42/Aβ40 at baseline adjusted for age, *APOE* ε4 status, and education carried an increased risk of progression (~70%) to AD dementia over 2 years (Perez‐Grijalba *et al*, 2019), with an AUC of 0.86 by comparison to stable MCI.

### Prediction of AD dementia and cognitive decline in CU

In longitudinal studies that have examined the association between plasma Aβ42/Aβ40 and the risk of progression to MCI or AD dementia in CU individuals using up to 6 years follow‐up, time‐dependent receiver operating characteristic curves—a method which takes into account interindividual differences in follow‐up and conversion times—showed that Simoa‐based plasma Aβ42/Aβ40 had AUC values ≥ 0.85 across all yearly time points (range 0.85–0.92) (Stockmann *et al*, 2020). Plasma Aβ42/Aβ40 has also been shown to be associated with progression to both MCI and AD dementia (Verberk *et al*, 2018, 2021), independent of potential confounders (education; *APOE* carriership; or medication use for hypertension, hypercholesterolemia, and diabetes) and measures of neuroinflammation and neurodegeneration (plasma GFAP and NfL). Lower (more abnormal) plasma Aβ42/Aβ40 has also been shown to relate to a more pronounced decline in composite cognitive scores over time (adjusted for sex, age, education, treatment group, BMI, CDR, GDS score) (Giudici *et al*, 2020). This finding also held when using longitudinal MMSE scores. Similar results have been reported using domain‐specific findings, including attention, memory, language, and executive functioning (Verberk *et al*, 2020a).

## Markers of tau pathology (P‐tau181, P‐tau217, and P‐tau231)

The identification of hyperphosphorylated tau as a major constituent of neurofibrillary tangles (Grundke‐Iqbal *et al*, 1986a, 1986b) led to the development of CSF assays for P‐tau (Iqbal & Grundke‐Iqbal, 1997) targeting specific serine and threonine amino acid residues. Though multiple phosphorylation sites exist on the tau protein (Portelius *et al*, 2008), the most commonly used assays for P‐tau detect phosphorylation at threonine 181 (P‐tau181). Using this measure, increased CSF P‐tau181 has been consistently shown in AD (Blennow *et al*, 2015). Using several different outcomes, however—including the separation of AD dementia from non‐AD neurodegenerative disorders and correlations with amyloid and tau PET—P‐tau217 has shown somewhat better performance than P‐tau181 (Janelidze *et al*, 2020b). Recently, P‐tau231 has also been detected in CSF (Buerger *et al*, 2003; Suarez‐Calvet *et al*, 2020) and plasma (Ashton *et al*, 2021c).

Although previous studies assumed that soluble P‐tau measures reflected tau pathology in AD (Jack *et al*, 2018), direct correlations between soluble P‐tau and neuropathology or PET measures of tau typically only found moderate correlations (Buerger *et al*, 2006, 2007; Mattsson *et al*, 2017b; La Joie *et al*, 2018).

More recent studies have instead linked changes in soluble P‐tau (for both CSF and plasma) to the accumulation of Aβ (Sato *et al*, 2018; Mattsson‐Carlgren *et al*, 2020a) and shown that changes in soluble P‐tau precede tau aggregation in AD as measured by PET or with neuropathology (Mattsson‐Carlgren *et al*, 2021).

### Assays

In contrast to CSF where commercial P‐tau181 assays target mid‐region forms of tau phosphorylated at threonine 181 (Vanderstichele *et al*, 2006; Leitao *et al*, 2019; Lifke *et al*, 2019), the development of assays for P‐tau in blood have focused on N‐terminal to mid‐region tau fragments following the discovery that this is the predominant tau species in blood (Sato *et al*, 2018). For instance, Tatebe and colleagues (Tatebe *et al*, 2017) developed a Simoa‐based plasma P‐tau181 assay by replacing the detection antibody in an existing T‐tau assay with a monoclonal antibody specific for P‐tau181. This study was the first to report elevated levels of plasma P‐tau181 in AD dementia, yet the assay suffered from insufficient analytical sensitivity. Using electrochemiluminescence (ECL)‐based methods developed by Eli Lilly, significant increases in plasma P‐tau181 and P‐tau217 have been reported in AD (Mielke *et al*, 2018; Janelidze *et al*, 2020a). Novel P‐tau181 and P‐tau217 assays targeting P‐tau isoforms containing the N‐terminal amino acid 6–18 epitope were then later developed (Karikari *et al*, 2020b, 2021). Though designed for use with blood, these assays also work for the quantification of P‐tau in CSF (Janelidze *et al*, 2020a; Karikari *et al*, 2020a; Suarez‐Calvet *et al*, 2020). Two additional Simoa‐based assays from Janssen targeting P‐tau217 and phosphorylation at amino acid 212 have also been recently described (Triana‐Baltzer *et al*, 2020, 2021) as well as a study providing a direct comparison of modified versions of the ECL‐based Eli Lilly assays for P‐tau217 and P‐tau181 (Thijssen *et al*, 2021a) that differed only in their epitope‐specific capture antibodies.

**Figure 1:**
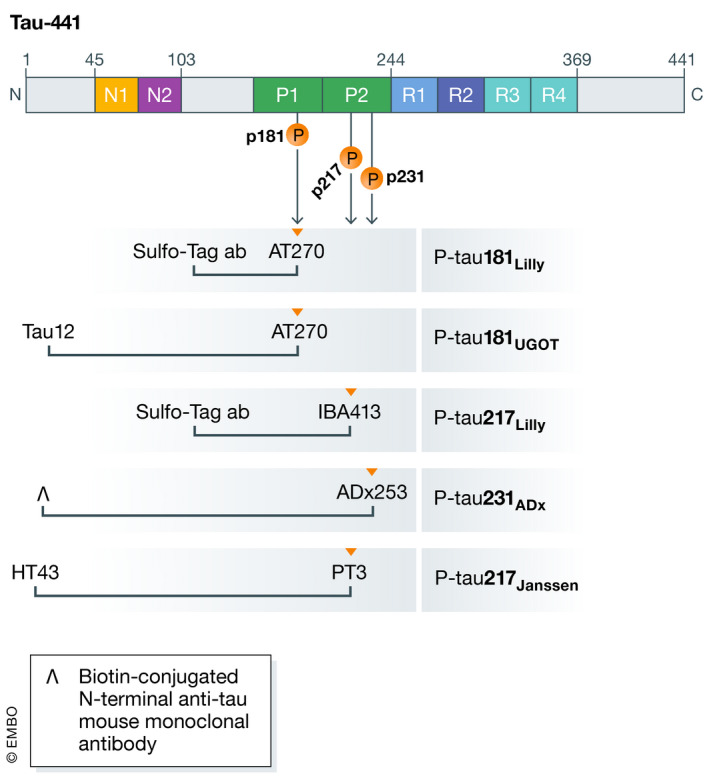
A schematic overview of the included P‐tau assays Schematic illustration of full‐length tau‐441, including N‐terminal, proline‐rich region, microtubuli binding domain, and C‐terminal. Anti‐tau antibodies are indicated for each of the five included P‐tau assays under the respective epitope region. P‐tau181_UGOT_ is the P‐tau181 assay from the University of Gothenburg, as detailed in Karikari *et al* (2021). For P‐tau231_ADx_, the inverted V symbol represents a biotin‐conjugated N‐terminal anti‐tau mouse monoclonal antibody, as detailed in Ashton *et al* (2021c).

Quantification of P‐tau231 in plasma has also recently been described using a phospho‐specific cis‐conformational monoclonal antibody ADx253 as a capture antibody and a biotin‐conjugated N‐terminal anti‐tau mouse monoclonal antibody for detection (Ashton *et al*, 2021c). A schematic overview of P‐tau assays is provided in Fig 1.

### Differential diagnosis of AD dementia

Increasingly, biomarkers are being incorporated into the clinical work‐up of patients presenting with cognitive impairment, in part due to the difficulty in differentiating AD from related non‐AD neurodegenerative disorders early on in the disease course (Beach *et al*, 2012; Salloway *et al*, 2014). In recent studies that have examined AD plasma biomarkers (Janelidze *et al*, 2020a; Karikari *et al*, 2020b; Palmqvist *et al*, 2020; Thijssen *et al*, 2020), diagnostic accuracies (AUC) for the separation of AD from non‐AD dementia disorders using clinical diagnosis as the standard of truth have ranged from 0.81 (Palmqvist *et al*, 2020) to 0.89 (Thijssen *et al*, 2020). In a larger cohort, plasma P‐tau181 was shown to differentiate AD dementia from non‐AD dementia disorders with a somewhat higher AUC (0.94), similar to those achieved using CSF P‐tau181 or tau PET (Janelidze *et al*, 2020a). More importantly, plasma P‐tau181 has been shown to be able to differentiate AD dementia from other neurodegenerative disorders using neuropathologically confirmed cases, with AUCs ranging from 0.85 (Janelidze *et al*, 2020a) to 0.95 (Thijssen *et al*, 2020). High accuracy for plasma P‐tau181 for discriminating AD from non‐pathologies when using neuropathological diagnoses as standard of truth was also reported (AUC of 0.97) using measures taken 8 years prior to autopsy (Lantero Rodriguez *et al*, 2020).

Using plasma P‐tau217, AD dementia cases were separated from non‐AD disorders with an AUC of 0.96 (Palmqvist *et al*, 2020) (Table EV1). Using neuropathologically confirmed cases, AUCs ranged from 0.89 to 0.98 (Palmqvist *et al*, 2020) for intermediate to high likelihood of AD, respectively (Mirra *et al*, 1991; 1997). In a recent head‐to‐head study comparing plasma P‐tau181 and P‐tau217 (Thijssen *et al*, 2021a)—measured using electrochemiluminescence‐based assays differing only in the biotinylated antibody epitope—though both measures showed high AUC values for differentiating pathology‐confirmed AD from pathologically confirmed FTLD, P‐tau217 slightly outperformed P‐tau181 (AUC 0.93 vs 0.91) when separating clinically diagnosed AD from FTLD syndromes. Plasma P‐tau231 has also been recently shown to have high accuracy for AD using clinical (AUC of 0.93) and neuropathological diagnoses (AUC of 0.99) using samples drawn 4.2 years on average prior to post‐mortem (Ashton *et al*, 2021c).

### Prediction of AD dementia and cognitive decline in MCI

In patients with MCI, baseline plasma P‐tau181 levels have been shown to be increased in those who progressed to AD dementia compared to those that did not develop dementia or that developed dementia due to other causes (Janelidze *et al*, 2020a; Therriault *et al*, 2021) (Table EV2). Plasma P‐tau181 levels were also elevated in Aβ‐positive MCI who progressed to AD dementia by comparison to both Aβ‐positive and Aβ‐negative CU and MCI who did not convert to AD dementia. After adjusting for age, sex, and education, higher baseline plasma P‐tau181 levels were associated with a greater risk of progression to AD dementia, with a hazard ratio nearly identical to that for CSF P‐tau181. Similar findings were obtained when adjusting plasma P‐tau181 for plasma T‐tau, Aβ42/Aβ40, and NfL (Janelidze *et al*, 2020a). In a related study, survival analysis showed that high baseline plasma P‐tau181 was associated with an increased risk of progression to AD dementia in MCI over 84 months, as compared with Aβ‐negative CU, with similar findings observed using a shorter follow‐up interval of 48 months (Karikari *et al*, 2021). Using both follow‐up intervals, the performance of plasma P‐tau181 was similar to that for CSF P‐tau181. Plasma P‐tau181 has also been shown to outperform other plasma biomarkers for separating MCI patients who converted to AD dementia from those that did not, though the AUC reported in this study was modest (0.77) (Simren *et al*, 2021).

Using plasma P‐tau217, higher baseline levels were observed in MCI patients who progressed to AD dementia (Mattsson‐Carlgren *et al*, 2020b). Further, plasma P‐tau217 levels were found to increase over time, as compared to stable P‐tau217 levels in MCI patients who did not develop AD dementia. In work addressing the association between longitudinal plasma P‐tau217 and longitudinal cognition in MCI (Mattsson‐Carlgren *et al*, 2020b), increases in plasma P‐tau217 were associated with worsening cognition. Similar findings have been reported for plasma P‐tau181 (Chen *et al*, 2021; Therriault *et al*, 2021).

### Prediction of AD dementia and cognitive decline in CU

In CU individuals, elevated baseline plasma P‐tau181 levels (adjusted for age, sex, and education) were found to carry an increased risk of progression to AD dementia (mean follow up of 4.9 years), with a hazard ratio nearly equivalent to that for CSF P‐tau181 (Janelidze *et al*, 2020a) (Table EV3). This finding held even after adjusting plasma P‐tau181 levels for plasma T‐tau, Aβ42/Aβ40, and NfL. High plasma P‐tau181 has also been shown to carry an increased risk of AD dementia in Aβ‐positive CU as compared with Aβ‐negative CU over 84 months and over a shorter interval of 48 months (Karikari *et al*, 2021). Here, however, the hazard ratio of CSF P‐tau181 was superior to that of plasma P‐tau181 (5.4 vs 3.25, respectively). Over a longer interval of 100 months, CU individuals who were plasma P‐tau181‐positive at baseline showed a faster cognitive decline compared to CU individuals who were P‐tau181‐negative (Karikari *et al*, 2021). Similar findings were shown over 60 months. Longitudinal increases in plasma P‐tau181 have also been found to be associated with future cognitive decline in CU individuals (Moscoso *et al*, 2021; Therriault *et al*, 2021). In a study examining the associations between longitudinal plasma P‐tau217 and longitudinal cognition (Mattsson‐Carlgren *et al*, 2020b), higher plasma P‐tau217 was associated with worsening cognition in CU individuals using both MMSE and mPACC as outcomes. In an analysis combining CU and MCI individuals (Pereira *et al*, 2021b), plasma P‐tau181 and P‐tau217 levels were associated with longitudinal decline in MMSE scores; however, P‐tau217 provided the best fit to the data and a larger effect size compared to P‐tau181. When combining plasma P‐tau181, P‐tau217, Aβ42/40, and NfL in a multivariate model, only P‐tau217 was a significant independent predictor of cognitive decline.

### Markers of neurodegeneration

Among fluid biomarkers of neurodegeneration, NfL is one of the most promising. A marker of subcortical large‐caliber axonal degeneration (Hoffman *et al*, 1987; Norgren *et al*, 2003), CSF levels of NfL are elevated in AD and ever more so in ALS, FTD, and atypical parkinsonian disorders (i.e., PSP, MSA, and CBD) (Khalil *et al*, 2018) and after acute brain injury (e.g., stroke, traumatic brain injury or cardiac arrest). Due it being increased in multiple neurological disorders, NfL is considered to be a nonspecific marker of neuronal injury (preferentially axonal injury). Importantly, higher levels of NfL are associated with faster disease progression and higher rates of brain atrophy in most neurodegenerative disorders (Khalil *et al*, 2018; Preische *et al*, 2019); as such, NfL can be regarded as a measure of the intensity of ongoing neurodegeneration. NfL can also be measured in blood, where levels are elevated in the prodromal (Mattsson *et al*, 2017a) and dementia stages (Mattsson *et al*, 2019) of sporadic AD as well as in autosomal dominant AD (Preische *et al*, 2019; Quiroz *et al*, 2020). Similar to CSF, however, plasma NfL is not specific for AD and is elevated in all neurodegenerative disorders, in particular FTD (Rohrer *et al*, 2016), ALS (Verde *et al*, 2019) and atypical parkinsonian disorders (Donker Kaat *et al*, 2018). Other blood‐based markers of neurodegeneration, including SB100 and T‐tau, have also been described in AD (Olsson *et al*, 2016; Michetti *et al*, 2019); the clinical relevance of these measures has been shown to be comparatively low, however, and as such will not be covered here.

### Assays

Plasma NfL has historically been measured using ELISA and ECL assay technology. However, the sensitivity of ELISA for quantifying plasma NfL has been found to be insufficient, and ECL is not sufficiently sensitive to detect the lowest concentrations of plasma NfL (Li & Mielke, 2019). Therefore, studies now utilize the ultra‐sensitive Simoa platform due to its greater sensitivity (Disanto *et al*, 2017; Gaetani *et al*, 2019). Plasma NfL concentrations in studies on AD (Mattsson *et al*, 2017a, 2019; Weston *et al*, 2017; Lewczuk *et al*, 2018; Mielke *et al*, 2019; Preische *et al*, 2019; Benedet *et al*, 2020) have since been measured on the Simoa platform (HD‐1 Analyser, Quanterix) (Gisslen *et al*, 2016; Rohrer *et al*, 2016; Disanto *et al*, 2017; Thijssen *et al*, 2021a).

### Differential diagnosis of AD dementia

Using plasma NfL, poor diagnostic performance has been reported for the separation of bvFTD patients from those with AD dementia (AUC of 0.68) (Steinacker *et al*, 2018) (Table EV1). Similar findings have been reported when separating AD dementia from a broader group of non‐AD disorders (AUC of 0.75), including bvFTD, non‐fluent/agrammatic variant of primary progressive aphasia (nfPPA), semantic variant of progressive aphasia (svPPA), progressive supranuclear palsy (PSP), corticobasal syndrome (CBS), and ALS‐FTD (Illan‐Gala *et al*, 2021). In another study (Palmqvist *et al*, 2020), plasma NfL had an AUC of 0.51 for separating participants with a high likelihood of AD (tangles in neocortex and moderate‐to‐frequent Aβ plaques) from non‐AD participants (those with none‐to‐sparse Aβ plaques) based on neuropathological assessment (Mirra *et al*, 1991) and 0.50 for clinical AD dementia vs other neurodegenerative disorders. In a recent study, plasma NfL showed an AUC of 0.82 for separating clinically diagnosed AD dementia from patients with FTLD syndromes (Thijssen *et al*, 2021a), and AUCs of 0.97 and 0.96 for distinguishing neuropathologically confirmed AD from neuropathologically confirmed FTLD‐tau and FTLD‐TDP cases, respectively. When examining plasma NfL's ability to differentiate AD dementia from various non‐AD neurodegenerative disorders in two multicenter cohorts (Ashton *et al*, 2021a), AUCs ranged from 0.53 (PD/PDD) to 0.88 (ALS).

### Prediction of AD dementia and cognitive decline in MCI

While those MCI patients with a positive Aβ‐biomarker have been shown to have higher plasma NfL levels than Aβ‐negative MCI, no differences in plasma NfL levels have been observed between Aβ‐positive patients who progressed to AD dementia after at least 2 years follow‐up and Aβ‐positive patients who did not (Mattsson *et al*, 2017a) (Table EV2). Elevated plasma NfL levels have also been associated with progression from MCI to all‐cause dementia over intervals of 2 and 4 years (Cullen *et al*, 2021a). Higher plasma NfL has also been associated with worse performance on several cognitive tests, including MMSE, ADAS‐COG 11, delayed recall of the WMS logical memory II, TMT‐B score, and WAIS‐R digit symbol substitution test score (Mattsson *et al*, 2017a), as well as a cognitive composite score across the cognitive domains of memory (free and total recall of the Free and Cued Selective Reminding Test [FCSRT]), language (the Category Naming Test), executive function (the DSST‐WAISR), and orientation (10 MMSE orientation items) (He *et al*, 2021).

Associations between increases in NfL and cognitive decline in MCI have also been reported using longitudinal data (Mattsson *et al*, 2019; He *et al*, 2021). In multivariable analyses combining MCI and CU individuals—adjusting for age, sex, and education—baseline plasma NfL was not found to be significantly associated with cross‐sectional cognitive measures (Mielke *et al*, 2019). Higher levels of baseline plasma NfL were significantly associated with declines in global cognition, however (Mielke *et al*, 2019). Higher baseline plasma NfL and longitudinal increases were both shown to associate with global cognition (Moscoso *et al*, 2021); this was reported in a clinically impaired group, however, though nearly three quarters had a diagnosis of MCI.

### Prediction of AD dementia and cognitive decline in CU

Elevated plasma NfL has also been shown to carry an increased risk of all‐cause dementia (de Wolf *et al*, 2020; Verberk *et al*, 2021) and dementia due to AD (Verberk *et al*, 2021) in large‐scale prospective population‐based and memory clinic‐based cohorts. Among CU individuals, cross‐sectional and longitudinal measures of plasma NfL have been shown not to be significantly associated with cognition (Mattsson *et al*, 2017a, 2019) (Table EV3). In multivariate analyses (adjusted for age, sex, and education) combining CU and MCI individuals, no significant associations were found between plasma NfL levels at baseline and cross‐sectional cognitive measures (Mielke *et al*, 2019). Significant associations were found, however, between higher baseline plasma NfL and longitudinal cognition (Mielke *et al*, 2019). Other work showed no significant associations between plasma NfL and cognitive decline (Moscoso *et al*, 2021) when accounting for plasma P‐tau181.

### Markers of neuroinflammation

Increasing evidence indicates that neuroinflammation plays an important role in AD and related dementia disorders (Calsolaro & Edison, 2016; Ardura‐Fabregat *et al*, 2017). Relevant biomarkers here include glial (astrocytic) proteins such as YKL‐40 (also known as chitinase‐3‐like‐1 protein), S100 calcium‐binding protein B (S100B), and glial fibrillary acidic protein (GFAP) (Carter *et al*, 2019). GFAP forms a major part of the cytoskeleton of astrocytes and is a marker of astroglia activation (Hol & Pekny, 2015). CSF levels of GFAP have been found to be significantly increased in AD and related neurodegenerative diseases compared to CU individuals (Ishiki *et al*, 2016; Abu‐Rumeileh *et al*, 2019). Plasma GFAP levels are elevated in CU older adults at risk of AD due to amyloid PET positivity (Chatterjee *et al*, 2021). As GFAP levels appear to increase in response to Aβ and tau aggregation (Garwood *et al*, 2017), GFAP will probably not be used as a marker of neuroinflammation but instead more often as a marker of Aβ.

### Assays

Studies examining the role of blood GFAP in AD have so far all used Simoa technology using the Human GFAP Discovery Kit from Quanterix (Lexington, MA, USA) (Oeckl *et al*, 2019; Cicognola *et al*, 2021; Pereira *et al*, 2021a; Verberk *et al*, 2021).

### Differential diagnosis of AD dementia

Using Simoa technology, significantly increased serum GFAP levels were observed in AD as compared to patients with bvFTD (Oeckl *et al*, 2019). Further, serum GFAP discriminated AD from bvFTD patients with 89% sensitivity and a specificity of 79% (Oeckl *et al*, 2019) (Table EV1).

### Prediction of AD dementia and cognitive decline in MCI and CU

When using progression from MCI to AD dementia as outcome, plasma GFAP had an AUC of 0.84 (Cicognola *et al*, 2021) (Table EV2). The addition of age and *APOE* ε4 did not improve diagnostic accuracy. MCI patients who converted to AD dementia also showed steeper slopes when looking at longitudinal GFAP, as compared to stable MCI (Cicognola *et al*, 2021). Serum GFAP levels have also been shown to predict future cognitive decline and progression to dementia in CU individuals (Verberk *et al*, 2021) (Table EV3). Plasma GFAP levels have also been shown to be associated with cognitive decline in a cohort combining individuals with and without cognitive impairment (Pereira *et al*, 2021a). No significant results were found when the analyses were conducted in the CU and CI groups separately, however.

## Diagnostic algorithms

Though disclosing a diagnosis of MCI is challenging for clinicians, patients, and their families (Whitehouse *et al*, 2004), findings suggest that the majority of people wish to be informed, particularly if AD is the underlying cause (Marzanski, 2000). In addition, having this information has been shown to change patient management (Rabinovici *et al*, 2019). Applying a multivariate approach to plasma biomarkers (Aβ42/Aβ40, P‐tau181, T‐tau, and NfL), Janelidze *et al* showed that P‐tau181 best predicted longitudinal conversion to AD dementia (Janelidze *et al*, 2020a) (Table EV4). Building on this work by applying the recent Aβ (A), tau (T), and neurodegeneration (N) classification system (Jack *et al*, 2016, 2018) to plasma AD biomarkers (Cullen *et al*, 2021a), a model combining P‐tau181 and NfL—but not Aβ42/Aβ40—was found to best predict cognitive decline (MMSE) and progression to AD dementia over a period of 4 years. Moreover, plasma‐based models showed either non‐inferiority or superior performance compared to corresponding CSF‐based models and outperformed a basic model consisting of age, sex, education, and baseline MMSE (Cullen *et al*, 2021a) (Table EV4). As a result of extensive internal and external validation analyses, the study by Cullen *et al* (2021a) provided an individualized online risk prediction tool for cognitive decline (MMSE, CDR‐SB, and conversion to AD dementia at 2 and 4 years after baseline) in patients with MCI (http://predictprogression.com). After entering age, sex, and baseline cognition (MMSE, CDR‐SB) into the tool, the user indicates biomarker status (positive or negative) for plasma (or CSF) Aβ42/Aβ40, P‐tau181, and NfL. The tool also allows for the testing of sparser models including the absence of biomarkers altogether or the availability of a subset only (http://predictprogression.com). The tool then returns linear and logistic regression model‐based prediction intervals and probabilities of conversion to AD dementia at 2 and 4 years after baseline.

When combining plasma biomarkers to predict cognitive decline in CU individuals (Cullen *et al*, 2021b), P‐tau217, Aβ42/Aβ40, and NfL together provided the most robust results. When examining progression to AD dementia, the combination of P‐tau217 and Aβ42/Aβ40 proved best, while P‐tau217, Aβ42/Aβ40, and NfL together proved best when examining conversion to all‐cause dementia (Cullen *et al*, 2021b) (Table EV4). Additional work has also shown that while high baseline GFAP and NfL were associated with an increased risk of all‐cause dementia, only GFAP remained significantly associated with an increased risk of dementia when both markers were entered together in the same model (Verberk *et al*, 2021). When adding plasma Aβ42/Aβ40 to this model, both GFAP and Aβ42/Aβ40 were independently associated with incident dementia, whereas NfL was not (Table EV4). When added to a model consisting of demographics, genetic, and clinical information (age, sex, education and *APOE ε4* genotype and a composite cardiovascular and metabolic conditions score), plasma P‐tau181 significantly improved prediction of memory decline (using both binary and continuous data) in CU and MCI individuals (Therriault *et al*, 2021).

Though diagnostic algorithms—and machine learning‐based approaches, more broadly—are ultimately intended to support personalized medicine, allowing for individualization at the levels of diagnosis, prognosis and treatment, proper oversight, and validation of such tools are crucial. This includes ensuring the absence of technical errors that can result in erroneous risk scores and inappropriate generalization from the training sample without robust validation (Handelman *et al*, 2018). Additional studies must also address how well these and other algorithms perform in predicting AD in primary care settings and require validation in large, unselected populations, including ethnically diverse groups due to the risk of the algorithm accuracy varying across certain subpopulations, such as racial and ethnic minority groups (Grote & Berens, 2020). In such studies, it is important that the biomarkers are analyzed prospectively in a consecutive manner using pre‐defined cut offs when appropriate.

## Clinical trials

For trial enrichment, blood‐based biomarkers can first help identify individuals with a high probability of having AD pathology—for instance, using plasma Aβ42/Aβ40 or P‐tau (Palmqvist *et al*, 2019b; Janelidze *et al*, 2020a). This approach is most likely needed in preclinical AD trials and may substantially reduce the number of negative CSF and PET examinations commonly seen during the screening phase for disease‐modifying trials (Cummings, 2019; Palmqvist *et al*, 2019b; Jack, 2020; Sperling *et al*, 2020). One possibility is that blood‐based biomarkers can be used as pre‐screening tools to select individuals for further, more definitive testing with CSF or PET examinations. This is likely to be the case when CSF or PET is being used to monitor pharmacodynamic treatment effects. In another possible scenario, if blood‐based biomarkers can achieve sufficiently high performance, they may be used without additional CSF or PET testing (but perhaps in combination with other modalities that are likely to be available anyway, such as magnetic resonance imaging (MRI) or cognitive tests, to boost their performance). This is suitable in trials where CSF or PET is not needed to subsequently show drug target engagement or pharmacodynamic response in every participant.

Blood‐based AD biomarkers could also be used as pharmacodynamic markers to detect direct target engagement and disease‐modifying effects. Both of these effects are critical to establish an appropriate dose and increase confidence that treatment results in disease‐relevant outcomes. If pharmacodynamic effects have less variability and changes that predict standard cognitive or functional outcomes, these blood‐based biomarkers could have utility as surrogate endpoints resulting in shorter clinical trials or smaller sample sizes. In the early stages of drug development, this may support go/no‐go decisions for subsequent larger trials of drug candidates, before effects are demonstrated on measures of cognition or function. To our knowledge, however, there are no published reports of results of putative AD disease‐modifying treatments (DMTs) on blood‐based biomarkers for Aβ or P‐tau. However, we note that there are reports of reduced CSF P‐tau levels after treatment with certain anti‐Aβ agents (Salloway *et al*, 2014; Ostrowitzki *et al*, 2017). Future studies will likely show whether similar effects are seen on blood‐based P‐tau measures.

Natural history studies have shown that plasma P‐tau has properties that may make it suitable as a pharmacodynamic marker, including longitudinal increases over a few years in both the preclinical and prodromal stages of AD, and longitudinal correlations with measures of cognition and brain structure (Mattsson‐Carlgren *et al*, 2020b). Changes in soluble P‐tau represent downstream effects from drug engagement of Aβ. However, we note that alterations in soluble P‐tau are closely linked to aggregated Aβ in the early stages of AD before there are increases in tau PET (Mattsson‐Carlgren *et al*, 2020a). It therefore seems unclear whether drug‐related reductions in aggregated Aβ will be followed quickly by changes in soluble P‐tau (in either CSF or plasma) and if soluble P‐tau will precede potential changes in tau PET by months or years. The strongest evidence that a drug affects the biological mechanisms of disease in a meaningful way may come from a general marker of neurodegeneration, such as NfL. Though there are as yet—to our knowledge—no published data on plasma NfL in trials of AD DMTs, decreases in plasma NfL levels have been shown to parallel clinical improvement in other neurological diseases, including in spinal muscular atrophy and multiple sclerosis (Kuhle *et al*, 2019; Olsson *et al*, 2019), where effective DMTs exist. This increases the likelihood that results for changes in plasma NfL may be robustly translated and interpreted between trials, independent of the primary drug target.

## Future directions and conclusions

**Figure 2:**
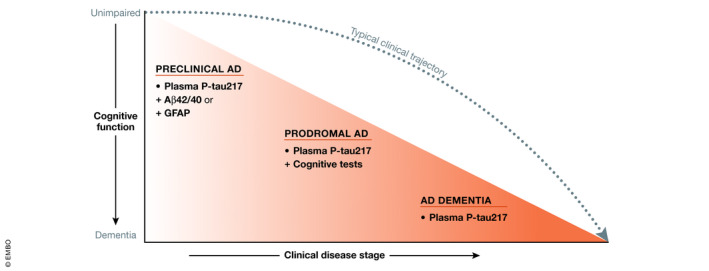
Most promising plasma‐based biomarkers across the clinical continuum of AD In this figure, cognitive function is represented on the *y*‐axis (top to bottom, unimpaired to dementia), with clinical disease stage shown on the *x*‐axis (preclinical AD, MCI due to AD [prodromal AD], and AD dementia). The dashed line represents the typical clinical trajectory from preclinical AD through prodromal and dementia stages. In each stage, the most promising plasma biomarker or plasma biomarker combination is indicated. For example, in preclinical AD, plasma P‐tau217 in combination with either plasma Aβ42/Aβ40 or GFAP is likely to prove best, while in prodromal AD plasma P‐tau217 in combination with simple to administer cognitive tests is likely best. In the AD dementia stage, plasma P‐tau217 alone is sufficient.

Rapid progress has been made in the field of blood‐based biomarkers for AD due to advances in sensitive and precise assays. Recent work (Ashton *et al*, 2021b; Boccardi *et al*, 2021) addressing the validation of AD blood biomarkers highlighted the need for studies addressing clinical validity and utility. Figure 2 provides a summary of the most promising plasma‐based biomarkers across the clinical continuum of AD. Much work remains, however, to develop and validate blood‐based biomarkers for non‐AD neurodegenerative disorders in order to be able to reliably measure key pathologies such as α‐synuclein or TDP‐43. From a clinical practice perspective, plasma biomarkers could be incorporated into primary care to determine which patients with cognitive symptoms have a high probability of having AD and should be referred to secondary settings for treatment and, when deemed necessary, also more advanced biomarker‐based investigations such as CSF or PET. However, in many countries, most elderly patients with cognitive symptoms are managed in primary care alone (i.e., not referred to secondary or tertiary centers). In a primary care setting, blood‐based diagnostic algorithms might already be sufficient to improve the accuracy of a clinical AD dementia diagnosis and positively influence patient management and care. Additional studies are required, however, to assess how to optimally combine plasma biomarkers, or other accessible and cost‐effective measures such as MRI and cognitive tests, and to further develop predictive algorithms, which will be especially important in non‐demented patients with cognitive complaints where additional non‐AD biomarkers could be incorporated. As described above, however, further validation is required in large ethnically diverse populations that are unselected and that have a lower pre‐test probability of underlying AD. In clinical trials, plasma biomarkers may be used to screen for individuals suitable for inclusion as well as disease‐relevant pharmacodynamic endpoint markers. Key requirements for the implementation of plasma biomarkers in these areas, however, are the development of high precision fully automated methods that can be used in clinical laboratory practice, the development of standard operating procedures for collecting and handling of blood samples prior to analyses, guidelines to ensure study reproducibility (Mattsson‐Carlgren *et al*, 2020c), and appropriate use criteria to guide clinical use, as has been done for CSF AD biomarkers (Shaw *et al*, 2018) and amyloid PET (Johnson *et al*, 2013).

## Author contributions

Drafting of the manuscript: AL, NM‐C, and OH. Critical revision of the manuscript for important intellectual content: AL, NM‐C, SJ, JLD, SP, and OH. Obtained funding and supervision.

## For more information

https://www.alzforum.org/alzbiomarker

**Pending issues**

- Much work remains to develop and validate blood‐based biomarkers that can reliably measure non‐AD pathologies such as α‐synuclein or TDP‐43.
- Studies are required in primary care settings to assess how best to combine plasma biomarkers with cognitive tests and other biomarkers and how to further develop algorithms capable of predicting cognitive decline and clinical progression.
- Validation studies on plasma biomarkers are required in large ethnically diverse populations that are unselected and have a lower pre‐test probability of underlying AD pathology.
- Overall, key requirements for the widespread use of plasma AD biomarkers include the development of high precision fully automated methods that can be used in clinical laboratory practice, standard operating procedures for the collection and handling of blood samples prior to analyses, reproducibility guidelines, and appropriate use criteria.

## Supporting information



Table EV1

Table EV2

Table EV3

Table EV4
